# Supplementary material for: Implementing Trauma-Informed Care—Settings, Definitions, Interventions, Measures, and Implementation across Settings: A Scoping Review
Source: Healthcare (Basel). 2024 Apr 27;12(9):908. doi: 10.3390/healthcare12090908 (PMC11083630; doi:10.3390/healthcare12090908)
Supplement: Supplementary file 1 [file healthcare-12-00908-s001.zip › healthcare-2861134-supplementary.pdf]

Supplement A

Overview of the included papers

Reference no.	Title	Year	Country [1]	Study design	Education	Other intervention	No intervention	Setting [2]	Sample Size	Org. level [3]	TIC Implementation checklist	Identification of Trauma
**[1]**	Successes and challenges in developing trauma-informed child welfare systems: A real-world case study of exploration and initial implementation	2017	1	Qualitative	Y	Y	N	1	658	2	Y	Y
**[2]**	Effects of integrated trauma treatment on outcomes in a Racially/Ethnically diverse sample of women in urban community-based substance abuse treatment	2007	1	Quantitative	Y	N	N	6	342	3	N	Y
**[3]**	Implementation of Trauma-Informed Care and Brief Solution-Focused Therapy: A Quality Improvement Project Aimed at Increasing Engagement on an Inpatient Psychiatric Unit.	2018	1	Quality Improvement	Y	N	N	2	33	5	N	N
**[4]**	Trauma-Informed Care in a Patient-Centered Medical Home for Adolescent Mothers and Their Children	2019	1	Quantitative	Y	N	N	3	429	4	N	Y
**[5]**	Restraint Reduction at a Pediatric Psychiatric Hospital: A Ten-Year Journey.	2015	1	Quality Improvement	Y	Y	N	1	844	4	N	N
**[6]**	Effectiveness of six core strategies based on trauma informed care in reducing seclusions and restraints at a child and adolescent psychiatric hospital	2017	1	Quantitative	Y	N	N	2	341	4	N	N

**[7]**	Development and psychometric evaluation of the Attitudes Related to Trauma-Informed Care (ARTIC) Scale	2016	1	Quantitative	N	N	Y	11	458	1	Y	N
**[8]**	Validation of the Attitudes Related to Trauma-Informed Care Scale (ARTIC)	2021	1	Quantitative	N	N	Y	11	1395	1	N	N
**[9]**	Integrating trauma-informed care and collective impact: perspectives of service providers working with cross-system youth	2021	1	Qualitative	Y	N	Y	11	35	2	N	N
**[10]**	Trauma-Informed Care in the Massachusetts Child Trauma Project	2015	1	Mixed Method	Y	N	N	1	760	2	Y	Y
**[11]**	The impact of a statewide trauma-informed child welfare initiative on children's permanency and maltreatment outcomes	2018	1	Quantitative	Y	Y	N	1	91253	2	N	N
**[12]**	Achieving Restraint-Free on an Inpatient Behavioral Health Unit	2009	1	Quantitative	Y	Y	N	2		5	N	N
**[13]**	Toward a trauma-informed state: An exploration of a training collaborative	2022	1	Mixed Method	N	N	N	1	2000 0	2	N	N
**[14]**	Trauma-Informed Care and Practice Practice Improvement Strategies in an Inpatient Mental Health Ward	2017	3	Quantitative	Y	N	N	2	26	5	N	Y
**[15]**	Changes in knowledge, beliefs, self-efficacy, and affective commitment to change following trauma-informed care education for pediatric service providers	2021	1	Quantitative	N	N	N	11	13	2	N	N
**[16]**	Trauma-informed attitudes in residential treatment settings: Staff, child and youth factors predicting adoption, maintenance and change over time	2021	2	Quantitative	Y	N	N	1	429	2	Y	N
**[17]**	Systematic investigation of initiatives to reduce seclusion and restraint in a state psychiatric hospital.	2011	1	Quantitative	Y	Y	N	2	27	4	Y	N
**[18]**	Frontline Staff Characteristics and Capacity for Trauma-Informed Care: Implications for the Child Welfare Workforce	2020	1	Quantitative	N	N	Y	1	271	2	Y	N
**[19]**	Psychometric evaluation of the TIC Grade, a self-report measure to assess youth perceptions	2022	1	Quantitative	N	N	N	7	95	2	Y	N

	of the quality of trauma-informed care they received											
**[20]**	Implementing trauma-informed care through social innovation in child welfare residential treatment centers serving elementary school children	2020	2	Quality Improvement	N	Y	N	1		2	Y	N
**[21]**	Risking Connection Trauma Training: A Pathway Toward Trauma-Informed Care in Child Congregate Care Settings	2012	1	Quantitative	Y	N	N	1	786	2	N	N
**[22]**	A trauma-informed approach to the medical history: teaching trauma-informed communication skills to first-year medical and dental students	2021	1	Quantitative	Y	N	N	4	164	4	N	N
**[23]**	Trauma Providers' Knowledge, Views, and Practice of Trauma-Informed Care	2018	1	Quantitative	N	N	Y	3	261	4	Y	N
**[24]**	Evaluating trauma informed care training for services supporting individuals experiencing homelessness and multiple disadvantage	2021	4	Quantitative	N	N	N	7	88	3	Y	N
**[25]**	Community ECHO [Extension for Community Healthcare Outcomes) Project Promotes Cross-Sector Collaboration and Evidence-Based Trauma-Informed â€¦	2021	1	Quantitative	N	N	N	11	100	2	N	Y
**[26]**	The Portal Project: A Layered Approach to Integrating Trauma into Alcohol and Other Drug Treatment for Women	2005	1	Mixed Method	N	Y	N	5	147	3	N	N
**[27]**	Trauma-informed education: creating and pilot testing a nursing curriculum on trauma-informed care	2020	1	Mixed Method	Y	N	N	4	151	6	Y	N
**[28]**	Providing trauma-informed care to women exiting prostitution: assessing programmatic responses to severe trauma	2019	1	Qualitative	N	N	Y	10	270	1	N	N
**[29]**	From traditional inpatient to trauma-informed treatment: transferring control from staff to patient.	2008	1	Qualitative	Y	Y	N	2	8	5	N	N
**[30]**	Reducing use of restraints and seclusion to create a culture of safety.	2012	1	Qualitative	N	N	N	2	10	5	N	Y

**[31]**	Pilot for Nurse-Led, Interprofessional In-Service Training on Trauma-Informed Perinatal Care	2015	1	Quantitative	Y	N	N	3	11	2	N	Y
**[32]**	Teaching trauma-informed care: a symposium for medical students	2020	1	Quality Improvement	Y	N	N	4	179	6	N	N
**[33]**	Developing a trauma-informed workforce for the opioid crisis in a rural community in the United States: a case study	2022	1	Mixed Method	N	N	N	11	115	2	N	N
**[34]**	In the trauma-informed care trenches: Teacher compassion satisfaction, secondary traumatic stress, burnout, and intent to leave education within underserved â€¦	2020	1	Quantitative	N	N	Y	9	163	3	Y	N
**[35]**	Changes in Treatment Content of Services During Trauma-informed Integrated Services for Women with Co-occurring Disorders	2009	1	Quantitative	Y	N	N	10	47	1	N	N
**[36]**	Psychiatric hospital nurses' attitudes towards trauma-informed care	2022	10	Mixed Method	N	N	Y	2	199	4	Y	N
**[37]**	Creating a communitywide system of trauma-informed care	2020	1	Quality Improvement	N	N	N	11	4000	3	N	N
**[38]**	Impacts of child welfare worker and clientele characteristics on attitudes toward trauma informed-care	2020	2	Quantitative	N	N	N	1	418	2	Y	N
**[39]**	Enhancing Capacity for Trauma-informed Care in Child Welfare: Impact of a Statewide Systems Change Initiative	2019	1	Quality Improvement	Y	N	N	1	2087	1	Y	Y
**[40]**	Trauma-informed care training in a child welfare system: Moving it to the front line	2013	1	Quantitative	Y	N	N	1	1651	2	Y	N
**[41]**	Promising practices and strategies for using trauma-informed child welfare practice to improve foster care placement stability: a breakthrough series collaborative.	2011	1	Quality Improvement	Y	Y	N	1	508	1	N	N
**[42]**	Trauma-informed children's ministry: A qualitative descriptive study	2021	1	Qualitative	N	N	N	10	64	1	N	N
**[43]**	Organizational and provider level factors in implementation of trauma-informed care after a city-wide training: an explanatory mixed methods assessment.	2017	1	Mixed Method	Y	Y	N	11	600	2	N	N
**[44]**	A mixed methods exploratory assessment of the usefulness of Baltimore City Health	2019	1	Mixed Method	Y	N	N	11	106	2	N	N

	Department's trauma-informed care training intervention.											
**[45]**	Trauma-informed practices to address intersections between HIV and intimate partner violence among women: perspective of community service providers	2020	2	Qualitative	N	N	N	7	12	2	N	N
**[46]**	Trauma-Informed Care Training in Family Medicine Residency Programs Results From a CERA Survey.	2018	1	Quantitative	N	N	Y	3	263	1	N	N
**[47]**	Trauma Talks: Exploring Personal Narratives of Trauma-Informed Care through Podcasting	2021	1	Qualitative	N	N	N	11	2	6	N	N
**[48]**	Reductions in behavioural and emotional difficulties from a specialist, trauma-informed school	2021	3	Quantitative	N	N	N	9	18	6	Y	Y
**[49]**	Child Welfare, Juvenile Justice, Mental Health, and Education Providers' Conceptualizations of Trauma-Informed Practice	2016	1	Qualitative	N	N	Y	11	126	2	N	N
**[50]**	Increasing Trauma-Informed Awareness and Practice in Higher Education	2020	1	Mixed Method	N	N	N	11	30	6	Y	N
**[51]**	Becoming trauma-informed: a case study of early educator professional development and organizational change	2021	1	Qualitative	N	Y	N	1	216	2	N	N
**[52]**	Advancing Trauma-Informed Systems Change in a Family Drug Treatment Court Context	2013	1	Qualitative	N	N	Y	5	12	3	N	Y
**[53]**	Training Pediatric Primary Care Residents in Trauma-Informed Care: A Feasibility Trial	2019	1	Mixed Method	Y	Y	N	4	33	4	N	Y
**[54]**	Trauma informed practices of a sober living home for women with addiction and victimization histories	2023	1	Quantitative	N	N	N	5	52	2	Y	Y
**[55]**	Importance of Leadership and Employee Engagement in Trauma-Informed Organizational Change at a Girlsâ€™ Juvenile Justice Facility	2017	1	Qualitative	Y	Y	N	2	17	5	N	N
**[56]**	Pilot Evaluation of K-12 School Security Professionals Online Training: Understanding Trauma and Social-Emotional Learning	2021	1	Quantitative	Y	N	Y	9	96	3	Y	N

**[57]**	Mind-Body Group for Teacher Stress: A Trauma-Informed Intervention Program	2019	1	Quality Improvement	Y	Y	N	9	16	3	N	N
**[58]**	Assessing Trauma-Informed Care Readiness in Behavioral Health: An Organizational Case Study	2011	1	Mixed Method	Y	N	N	2	145	2	N	N
**[59]**	A Pilot Study of a Trauma Training for Healthcare Workers Serving Refugees in Greece: Perceptions of Feasibility of Task-Shifting Trauma Informed Care	2020	8	Mixed Method	Y	N	N	3	33	2	N	N
**[60]**	Implementation of a workforce initiative to build trauma-informed child welfare practice and services: Findings from the Massachusetts Child Trauma Project	2014	1	Qualitative	Y	Y	N	1	192	1	Y	Y
**[61]**	Attitudes towards trauma-informed care in residential out-of-home care	2020	3	Quantitative	Y	N	N	7	55	3	Y	N
**[62]**	Developing trauma resilient communities through community capacity-building	2021	1	Quantitative	N	Y	N	11	1031	2	Y	N
**[63]**	A change in culture: violence prevention in an acute behavioral health setting.	2012	1	Quality Improvement	Y	N	N	2	48	5	N	Y
**[64]**	Medical Students' Perspectives on Trauma-Informed Care Training.	2018	1	Qualitative	Y	N	N	4	20	6	N	Y
**[65]**	Teaching trauma-related insight improves attitudes and behaviors toward challenging clients	2008	13	Quantitative	Y	N	N	3	303	1	N	N
**[66]**	What Does it Mean to be Trauma-Informed? A Multi-System Perspective from Practitioners Serving the Community	2021	1	Mixed Method	N	N	Y	11	24	3	Y	Y
**[67]**	Evidence-Based Practice: Implementing Trauma-Informed Care of Children and Adolescents in the Inpatient Psychiatric Setting	2023	1	Quality Improvement	Y	N	N	2		4	N	N
**[68]**	Educating emergency department nurses about trauma informed care for people presenting with mental health crisis: a pilot study	2016	3	Mixed Method	Y	N	N	3	34	4	N	N
**[69]**	Trauma-Informed Acute Care of Patients With Violence-Related Injury	2022	1	Qualitative	N	N	N	3	13	4	N	N
**[70]**	Introducing a trauma-□informed capability approach in youth services	2020	4	Mixed Method	N	N	Y	11	49	2	N	N

**[71]**	Development and Implementation of Trauma-Informed Programming in Youth Residential Treatment Centers Using the ARC Framework	2013	1	Quantitative	Y	Y	N	5	126	2	Y	Y
**[72]**	Experiences of lay social workers trained in a trauma-informed intervention in the deinstitutionalization of Rwanda	2021	11	Qualitative	N	N	N	10	10	2	N	N
**[73]**	Capacity Building for Refugee Mental Health in Resettlement: Implementation and Evaluation of Cross-Cultural Trauma-Informed Care Training	2020	1	Mixed Method	N	Y	N	11	175	1	N	N
**[74]**	Working towards Culturally Responsive Trauma-Informed Care in the Refugee Resettlement Process: Qualitative Inquiry with Refugee-Serving Professionals in the United States	2021	1	Qualitative	N	N	Y	11	78	2	N	N
**[75]**	Using trauma informed care as a nursing model of care in an acute inpatient mental health unit: A practice development process	2017	3	Qualitative	N	N	Y	2	5	5	N	N
**[76]**	Safe and Collaborative Communication Skills: A Step towards Mental Health Nurses Implementing Trauma Informed Care	2017	3	Mixed Method	Y	N	N	2	73	4	N	Y
**[77]**	Effectiveness of a Trauma-Informed Care Initiative in a State Child Welfare System: A Randomized Study	2019	1	Quantitative	Y	N	N	1	145	2	N	Y
**[78]**	Trauma-informed Day Services for Individuals with Intellectual/Developmental Disabilities: Exploring Staff Understanding and Perception within an Innovative Programme	2016	1	Qualitative	Y	N	N	8	20	5	N	N
**[79]**	Trauma-informed day services: An initial conceptualization and preliminary assessment	2017	1	Mixed Method	Y	N	N	8	28	4	Y	N
**[80]**	From the DSP Perspective: Exploring the Use of Practices That Align With Trauma-Informed Care in Organizations Serving People With Intellectual and Developmental Disabilities	2020	1	Quantitative	N	N	N	8	380	1	Y	N
**[81]**	Promoting Satisfaction and Reducing Fatigue: Understanding the Impact of Trauma-Informed Organizational Culture on Psychological Wellness among Direct Service Providers	2020	1	Quantitative	N	N	Y	8	380	2	Y	N

**[82]**	Implementation and program evaluation of trauma-informed care training across state child advocacy centers: An exploratory study	2017	1	Quantitative	Y	N	N	1	203	2	Y	Y
**[83]**	Development and Implementation of a Child Welfare Workforce Strategy to Build a Trauma-Informed System of Support for Foster Care	2016	1	Quantitative	Y	N	N	1	44	2	N	Y
**[84]**	Impact of Trauma-Informed Training and Mindfulness-Based Social-Emotional Learning Program on Teacher Attitudes and Burnout: A Mixed-Methods Study	2021	2	Mixed Method	N	N	N	9	112	3	N	Y
**[85]**	Organizational assessment to implement trauma-informed care for first responders, child welfare providers, and healthcare professionals	2021	1	Quantitative	Y	N	Y	1	327	3	N	N
**[86]**	How a shared humanity model can improve provider well-being and client care: An evaluation of Fraser Health's Trauma and Resiliency Informed Practice (TRIP) training program	2021	2	Mixed Method	N	N	N	3	62	2	N	Y
**[87]**	A patient-oriented research approach to assessing patients' and primary care physicians' opinions on trauma-informed care	2021	2	Quantitative	Y	N	Y	3	543	1	Y	N
**[88]**	A statewide introduction of trauma-informed care in a child welfare system	2012	1	Quantitative	Y	N	N	1	102	2	Y	Y
**[89]**	Sanctuary in a residential treatment center: creating a therapeutic community of hope countering violence	2016	1	Qualitative	Y	N	Y	1	23	4	N	Y
**[90]**	Sustaining interdisciplinary work in trauma-informed education	2022	3	Qualitative	Y	N	N	9	2	2	N	N
**[91]**	Installing trauma-informed care through the Tennessee Child Protective Services Academy.	2019	1	Quality Improvement	Y	Y	N	1	297	2	N	Y
**[92]**	Infusion of Trauma-Informed Care in Organizations: Experience of Agency Staff	2015	1	Quantitative	Y	N	Y	7	282	2	Y	N
**[93]**	Client Experiences of Trauma-Informed Care in Social Service Agencies	2018	1	Qualitative	Y	Y	Y	11	26	2	N	N
**[94]**	Building Capacity for Trauma-Informed Care in the Child Welfare System	2016	1	Quality Improvement	Y	N	N	1	454	2	Y	Y

**[95]**	Creating conditions for professional development through a trauma-informed and restorative practice	2022	6	Mixed Method	N	N	N	4	29	5	N	N
**[96]**	"We went as a team closer to the truth": impacts of interprofessional education on trauma- and violence- informed care for staff in primary care settings	2021	2	Qualitative	Y	N	Y	11	14	2	N	N
**[97]**	Trauma-informed care training for educators: some preliminary evidence	2020	1	Quantitative	Y	N	N	9	570	3	Y	N
**[98]**	Implementing Trauma-Informed Strategies for Mothers of Infants with Neonatal Abstinence Syndrome	2021	1	Quality Improvement	N	N	N	3	49	5	N	N
**[99]**	Implementing a Trauma-Informed Public Health System in San Francisco, California	2018	1	Quality Improvement	Y	N	Y	11	9000	2	N	N
**[100]**	Knowledge, Skills, and Self-Reflection: Linking Trauma Training Content to Trauma-Informed Attitudes and Stress in Preschool Teachers and Staff	2021	1	Quantitative	Y	N	Y	9	111	2	Y	Y
**[101]**	"Learning how to ask": Effectiveness of a training for trauma inquiry and response in substance use disorder healthcare professionals	2018	5	Quantitative	Y	N	N	6	74	2	N	N
**[102]**	Reducing barriers to trauma inquiry in substance use disorder treatment - a cluster-randomized controlled trial	2019	5	Quantitative	Y	Y	N	6	148	2	N	Y
**[103]**	Learning How to Ask-Does a one-day training increase trauma inquiry in routine substance use disorder practice? Results of a cluster-randomized controlled trial	2019	5	Quantitative	N	Y	N	6	2322	2	N	N
**[104]**	A science of hope? Tracing emergent entanglements between the biology of early life adversity, trauma-informed care, and restorative justice	2021	5	Qualitative	Y	N	N	11	15	2	N	N
**[105]**	An Evaluation of Whole-School Trauma-Informed Training Intervention Among Post-Primary School Personnel: A Mixed Methods Study	2022	4	Mixed Method	N	N	N	9	216	2	Y	Y
**[106]**	Evaluation of Trauma-Informed Integrated Health Models of Care for Women: A Qualitative Case Study Approach	2018	2	Qualitative	N	Y	N	5	39	2	N	N

**[107]**	Implementing trauma-informed care at a non-profit human service agency in Alaska: assessing knowledge, attitudes, and readiness for change.	2018	1	Quantitative	N	N	Y	7	61	2	N	N
**[108]**	Patterns of change in restraints, seclusions and time-outs over the implementation of trauma-informed staff training programs in residential care for children and youth	2022	2	Quantitative	Y	N	N	1	110	2	Y	N
**[109]**	Sensory modulation and trauma-informed-care knowledge transfer and translation in mental health services in Victoria: Evaluation of a statewide train-the-trainer intervention	2017	3	Qualitative	Y	Y	N	2	170	2	N	Y
**[110]**	Evaluating Foundational Professional Development Training for Trauma-Informed Approaches in Schools	2018	1	Quantitative	Y	N	N	9	183	2	N	N
**[111]**	Training Hospital Personnel in Trauma-Informed Care: Assessing an Interprofessional Workshop With Patients as Teachers	2021	1	Quantitative	Y	N	N	3	318	4	N	N
**[112]**	The Protective Role of Trauma Informed Attitudes on Perceived Stress Among Teachers and School Staff	2021	1	Quantitative	N	N	Y	9	396	3	Y	Y
**[113]**	Resilience Building Programs in U.S. Corrections Facilities: An Evaluation of Trauma-Informed Practices in Place	2021	1	Qualitative	Y	N	N	10	12	2	N	N
**[114]**	Trauma-informed child welfare systems and children's well-being: A longitudinal evaluation of KVC's bridging the way home initiative	2017	1	Quantitative	Y	N	N	1	1499	2	N	Y
**[115]**	Implementing Introductory Training in Trauma-Informed Care Into Mental Health Rehabilitation Services: A Mixed Methods Evaluation	2022	3	Mixed Method	N	N	N	2	131	2	Y	N
**[116]**	Effectiveness of 1-day trauma-informed care training programme on attitudes in psychiatric hospitals: A pre-post study	2019	9	Quantitative	Y	N	N	2	65	4	N	N
**[117]**	Examining the effectiveness of Trauma SmartÂ® training: Staff satisfaction, knowledge, and attitudes	2021	1	Quantitative	Y	N	N	9	2418	1	Y	N
**[118]**	Achieving Service Change Through the Implementation of a Trauma-Informed Care Training Program Within a Mental Health Service	2018	3	Quantitative	Y	N	N	11	121	2	N	N

**[119]**	Restorative Parenting: Delivering Trauma-Informed Residential Care for Children in Care	2021	4	Mixed Method	N	N	N	1	32	2	N	Y
**[120]**	Application of the Consolidated Framework for Implementation Research to Facilitate Delivery of Trauma-Informed HIV Care	2021	1	Mixed Method	N	N	Y	3	23	3	N	N
**[121]**	Exploring How Sexual Assault Nurse Examiners Practise Trauma-Informed Care	2021	2	Qualitative	N	N	Y	3	8	2	N	N
**[122]**	Cluster-randomized controlled trial of reducing seclusion and restraint in secured care of men with schizophrenia	2013	7	Quantitative	Y	Y	N	2	88	4	N	Y
**[123]**	Feasibility and Impact of Trauma-Informed Care Training in Internal Medicine Residency: A Pilot Study	2022	1	Quantitative	N	N	N	4	37	4	N	N
**[124]**	Planning for Youth Emotional Health in Unruly Environments: Bringing a Trauma Informed Community Building Lens to Therapeutic Planning	2020	1	Mixed Method	Y	N	Y	1		3	N	N
**[125]**	Promoting Wellness and Recovery of Young Women Experiencing Gender-Based Violence and Homelessness: The Role of Trauma-Informed Health Promotion Interventions	2021	2	Qualitative	Y	N	N	7	18	5	N	N
**[126]**	Implementation of the Six Core Strategies for Restraint Minimization in a Specialized Mental Health Organization	2016	2	Quantitative	Y	N	N	2	332	4	N	N
**[127]**	Improving outcomes for trauma-experienced individuals through the delivery of trauma awareness training for multi-organisational public sector workers	2022	4	Mixed Method	N	N	N	11	224	2	N	N
**[128]**	Understanding Staff- and System-Level Contextual Factors Relevant to Trauma-Informed Care Implementation	2021	1	Mixed Method	N	N	N	11	760	1	N	N
**[129]**	Initial Teacher Education and Trauma and Violence Informed Care in the Classroom: Preliminary Results from an Online Teacher Education Course	2020	2	Quantitative	Y	N	Y	10	287	2	Y	N
**[130]**	Exploratory study on the role of trauma-informed self-care on child welfare workers' mental health	2019	1	Quantitative	N	N	Y	1	184	5	N	N
**[131]**	Trauma-Informed Care and Health Among LGBTQ Intimate Partner Violence Survivors	2021	1	Quantitative	N	N	Y	3	239	1	Y	Y

**[132]**	Effect of trauma-informed care on hair cortisol concentration in youth welfare staff and client physical aggression towards staff: results of a longitudinal study	2020	12	Quantitative	N	N	N	1	142	2	N	N
**[133]**	Well-being of Latina survivors of intimate partner violence and sexual assault receiving trauma-informed and Culturally-Specific services	2020	1	Quantitative	Y	N	Y	7	175	1	Y	N
**[134]**	Trauma-informed primary care for medical residents.	2020	1	Quantitative	Y	N	N	4	21	4	N	N
**[135]**	A multi-dimensional conceptual framework for trauma-informed practice in addictions programming	2017	2	Qualitative	N	N	Y	5	41	3	N	N
**[136]**	Trauma-Informed Organizational Dynamics and Client Outcomes in Concurrent Disorder Treatment	2022	2	Qualitative	N	N	N	5	172	2	Y	N
**[137]**	Understanding the meaning of trauma-informed care for burns health care professionals in a pediatric hospital: A qualitative study using interpretive â€¦	2022	3	Qualitative	Y	N	N	3	20	4	N	N
**[138]**	How do staff in residential care transform Trauma-Informed principles into practice? A qualitative study from a Norwegian child welfare context	2022	6	Qualitative	N	N	N	1	19	3	N	N
**[139]**	Perceived lack of training moderates relationship between healthcare providers' personality and sense of efficacy in trauma-informed care.	2019	1	Quantitative	N	N	Y	3	172	2	N	N
**[140]**	Trust-Based Relational Intervention as a Trauma-Informed Teaching Approach	2021	1	Mixed Method	N	N	N	9	53	3	N	Y
**[141]**	Using the ARTIC-35 to Measure Nurses' Attitudes Related to Trauma-Informed Care	2020	2	Quantitative	N	N	N	11	274	1	Y	N
**[142]**	Consideration of Personal Adverse Childhood Experiences during Implementation of Trauma-Informed Care Curriculum in Graduate Health Programs.	2017	1	Quantitative	N	N	N	4	967	2	N	N
**[143]**	Evaluation of the effects of receiving trauma-informed practices on domestic violence shelter residents	2017	1	Quantitative	N	N	Y	5	57	2	N	N

**[144]**	Knowledge, principal support, self-efficacy, and beliefs predict commitment to trauma-informed care	2019	1	Quantitative	N	N	Y	11	118	2	N	Y
**[145]**	Delivering trauma-informed treatment in a women-only residential rehabilitation service: Qualitative study	2016	4	Qualitative	N	N	Y	5	37	3	N	N
**[146]**	Trauma-responsive child welfare services: A mixed methods study assessing safety, stability, and permanency	2019	1	Mixed Method	N	Y	N	1	59810	4	N	Y
**[147]**	Trauma-informed care: A qualitative study exploring the views and experiences of professionals in specialist health services for adults with intellectual disabilities	2019	4	Qualitative	N	N	Y	11	25	1	N	N
**[148]**	Organizational Trauma-Informed Care: Associations With Individual and Agency Factors	2019	1	Quantitative	N	N	Y	11	345	1	Y	N
**[149]**	Trauma-informed care training with HIV and related community service workers: Short and long term effects on attitudes	2021	2	Mixed Method	Y	N	N	7	150	2	Y	N
**[150]**	Using Participatory Methods to Engage Multidisciplinary Clinical Staff in the Embedding of Trauma-Informed Care and Practice Principles in a Sub-Acute Mental Health Inpatient Unit	2020	3	Quality Improvement	Y	N	N	2	24	5	N	N
**[151]**	Application of a Framework to Implement Trauma-Informed Care Throughout a Pediatric Health Care Network	2017	1	Quality Improvement	Y	N	N	3	440	4	N	N
**[152]**	The Development of Trauma and Resilience Competencies for Nursing Education	2021	1	Quality Improvement	N	N	N	4	16	6	N	N
**[153]**	Does training change practice? A survey of clinicians and managers one year after training in trauma-informed care	2017	3	Quality Improvement	Y	N	Y	2	271	2	N	N
**[154]**	Evaluating the Impact of Trauma-Informed Care (TIC] Perspective in Social Work Curriculum	2016	1	Quality Improvement				4	23	6	N	N
**[155]**	The New York State Office of Mental Health Positive Alternatives to Restraint and Seclusion (PARS] Project	2015	1	Quality Improvement	Y	N	N	1	64	2	N	N
**[156]**	Perceptions of We Can! Building Relationships and Resilience: A School Wide Trauma-	2022	1	Qualitative	Y	N	Y	9	75	2	N	N

	Informed Training, from Trainers and Recipients											
**[157]**	"The glue that makes the glitter stick": Preliminary outcomes associated with a trauma-informed, resiliency-based, interprofessional graduate course for child welfare, mental health, and education	2019	1	Quantitative	Y	N	N	1	40	2	N	N

Note. Missing=Not stated, described or actual in the paper.

1, Country: 1=USA, 2= Canada, 3=Australia, 4=UK, 5=Germany, 6=Norway, 7=Finland, 8=Greece, 9=Japan, 10=Malta, 11=Rwanda, 12=Switzerland, 13=Other

2, Setting: 1=Child Welfare, 2=Mental and behavioral Health, 3=Healthcare, 4=Healthcare education and Social work education, 5=Residential Treatment Programs, 6=Substance Abuse Services, 7=Social Services, 8=Intellectual and developmental disability, 9=School/child education, 10=Other, 11=Cross Sectoral

3, Organizational Level: 1=Country, 2=Region, 3=County/City, 4=Hospital, 5=Ward, 6=Social Work or Health Care Education School or University

Reference

1. Akin, B.A.; Strolin-Goltzman, J.; Collins-Camargo, C. Successes and Challenges in Developing Trauma-Informed Child Welfare Systems: A Real-World Case Study of Exploration and Initial Implementation. *Child. Youth Serv. Rev.* **2017**, *82*, 42–52, doi:10.1016/j.childyouth.2017.09.007.
2. Amaro, H.; Dai, J.; Arévalo, S.; Acevedo, A.; Matsumoto, A.; Nieves, R.; Prado, G. Effects of Integrated Trauma Treatment on Outcomes in a Racially/Ethnically Diverse Sample of Women in Urban Community-Based Substance Abuse Treatment. *J. Urban Health* **2007**, *84*, 508–522, doi:10.1007/s11524-007-9160-z.
3. Aremu, B.; Hill, P.D.; McNeal, J.M.; Petersen, M.A.; Swanberg, D.; Delaney, K.R. Implementation of Trauma-Informed Care and Brief Solution-Focused Therapy: A Quality Improvement Project Aimed at Increasing Engagement on an Inpatient Psychiatric Unit. *J. Psychosoc. Nurs. Ment. Health Serv.* **2018**, *56*, 16–22, doi:10.3928/02793695-20180305-02.
4. Ashby, B.D.; Ehmer, A.C.; Scott, S.M. Trauma-Informed Care in a Patient-Centered Medical Home for Adolescent Mothers and Their Children. *Psychol. Serv.* **2019**, *16*, 67–74, doi:10.1037/ser0000315.
5. Azeem, M.W.; Reddy, B.; Wudarsky, M.; Carabetta, L.; Gregory, F.; Sarofin, M. Restraint Reduction at a Pediatric Psychiatric Hospital: A Ten-Year Journey. *J. Child Adolesc. Psychiatr. Nurs.* **2015**, *28*, 180–184, doi:10.1111/jcap.12127.
6. Azeem, M.; Aujla, A.; Rammerth, M.; Binsfeld, G.; Jones, R.B. Effectiveness of Six Core Strategies Based on Trauma Informed Care in Reducing Seclusions and Restraints at a Child and Adolescent Psychiatric Hospital. *J. Child Adolesc. Psychiatr. Nurs.* **2017**, *30*, 170–174, doi:10.1111/jcap.12190.
7. Baker, C.N.; Brown, S.M.; Wilcox, P.D.; Overstreet, S.; Arora, P. Development and Psychometric Evaluation of the Attitudes Related to Trauma-Informed Care (ARTIC) Scale. *School Ment. Health* **2016**, *8*, 61–76, doi:10.1007/s12310-015-9161-0.
8. Baker, C.N.; Brown, S.M.; Overstreet, S.; Wilcox, P.D.; New Orleans Trauma-Informed Schools Learning Collaborative Validation of the Attitudes Related to Trauma-Informed Care Scale (ARTIC). *Psychol. Trauma Theory Res. Pract. Policy* **2021**, *13*, 505–513, doi:10.1037/tra0000989.
9. Ball, A.; Bowen, E.A.; Jones, A.S. Integrating Trauma-Informed Care and Collective Impact: Perspectives of Service Providers Working with Cross-System Youth. *J. Soc. Soc. Work Res.* **2021**, *12*, 59–81, doi:10.1086/712960.
10. Bartlett, J.D.; Barto, B.; Griffin, J.L.; Fraser, J.G.; Hodgdon, H.; Bodian, R. Trauma-Informed Care in the Massachusetts Child Trauma Project. *Child Maltreat.* **2016**, *21*, 101–112, doi:10.1177/1077559515615700.
11. Barto, B.; Bartlett, J.D.; Von Ende, A.; Bodian, R.; Noroña, C.R.; Griffin, J.; Fraser, J.G.; Kinniburgh, K.; Spinazzola, J.; Montagna, C.; et al. The Impact of a Statewide Trauma-Informed Child Welfare Initiative on Children's Permanency and Maltreatment Outcomes. *Child Abuse Negl.* **2018**, *81*, 149–160, doi:10.1016/j.chiabu.2018.04.023.
12. Barton, S.A.; Johnson, M.R.; Price, L.V. Achieving Restraint-Free on an Inpatient Behavioral Health Unit. *J. Psychosoc. Nurs. Ment. Health Serv.* **2009**, *47*, 34–40, doi:10.3928/02793695-20090101-01.
13. Beck, E.; Carmichael, D.; Blanton, S.; Bride, B.; Mobley, A.; DiGirolamo, A. Toward a Trauma-Informed State: An Exploration of a Training Collaborative. *Traumatology* **2022**, *28*, 471–479, doi:10.1037/trm0000351.
14. Beckett, P.; Holmes, D.; Phipps, M.; Patton, D.; Molloy, L. Trauma-Informed Care and Practice: Practice Improvement Strategies in an Inpatient Mental Health Ward. *J. Psychosoc. Nurs. Ment. Health Serv.* **2017**, *55*, 34–38, doi:10.3928/02793695-20170818-03.
15. Berg-Poppe, P.; Anis Abdellatif, M.; Cerny, S.; LaPlante, K.; Merrigan, M.; Wesner, C. Changes in Knowledge, Beliefs, Self-Efficacy, and Affective Commitment to Change Following Trauma-Informed Care Education for Pediatric Service Providers. *Psychol. Trauma Theory Res. Pract. Policy* **2022**, *14*, 535–544, doi:10.1037/tra0001083.

16. Black, K.R.; Collin-Vézina, D.; Brend, D.; Romano, E. Trauma-Informed Attitudes in Residential Treatment Settings: Staff, Child and Youth Factors Predicting Adoption, Maintenance and Change over Time. *Child Abuse Negl.* **2022**, *130*, 105361, doi:10.1016/j.chiabu.2021.105361.
17. Borckardt, J.J.; Madan, A.; Grubaugh, A.L.; Danielson, C.K.; Pelic, C.G.; Hardesty, S.J.; Hanson, R.; Herbert, J.; Cooney, H.; Benson, A.; et al. Systematic Investigation of Initiatives to Reduce Seclusion and Restraint in a State Psychiatric Hospital. *Psychiatr. Serv.* **2011**, *62*, 477–483, doi:10.1176/ps.62.5.pss6205_0477.
18. Bosk, E.A.; Williams-Butler, A.; Ruisard, D.; MacKenzie, M.J. Frontline Staff Characteristics and Capacity for Trauma-Informed Care: Implications for the Child Welfare Workforce. *Child Abuse Negl.* **2020**, *110*, 104536, doi:10.1016/j.chiabu.2020.104536.
19. Boucher, N.; Darling-Fisher, C.S.; Sinko, L.; Beck, D.; Granner, J.; Seng, J. Psychometric Evaluation of the TIC Grade, a Self-Report Measure to Assess Youth Perceptions of the Quality of Trauma-Informed Care They Received. *J. Am. Psychiatr. Nurses Assoc.* **2022**, *28*, 319–325, doi:10.1177/1078390320953896.
20. Brend, D.; Fréchette, N.; Milord-Nadon, A.; Harbinson, T.; Colin-Vezina, D. Implementing Trauma-Informed Care through Social Innovation in Residential Care Facilities Serving Elementary School Children. *Int. J. Child Adolesc. Resil.* **2020**, *7*, 222–232, doi:10.7202/1072600ar.
21. Brown, S.M.; Baker, C.N.; Wilcox, P. Risking Connection Trauma Training: A Pathway toward Trauma-Informed Care in Child Congregate Care Settings. *Psychol. Trauma Theory Res. Pract. Policy* **2012**, *4*, 507–515, doi:10.1037/a0025269.
22. Brown, T.; Mehta, P.K.; Berman, S.; McDaniel, K.; Radford, C.; Lewis-O'Connor, A.; Grossman, S.; Potter, J.; Hirsh, D.A.; Woo, B.; et al. A Trauma-Informed Approach to the Medical History: Teaching Trauma-Informed Communication Skills to First-Year Medical and Dental Students. *MedEdPORTAL* **2021**, 11160, doi:10.15766/mep_2374-8265.11160.
23. Bruce, M.M.; Kassam-Adams, N.; Rogers, M.; Anderson, K.M.; Sluys, K.P.; Richmond, T.S. Trauma Providers' Knowledge, Views, and Practice of Trauma-Informed Care. *J. Trauma Nurs.* **2018**, *25*, 131–138, doi:10.1097/JTN.0000000000000356.
24. Burge, R.; Tickle, A.; Moghaddam, N. Evaluating Trauma Informed Care Training for Services Supporting Individuals Experiencing Homelessness and Multiple Disadvantage. *Hous. Care Support* **2021**, *24*, 14–25, doi:10.1108/HCS-01-2021-0002.
25. Buysse, C.A.; Bentley, B.; Baer, L.G.; Feldman, H.M. *Community ECHO (Extension for Community Healthcare Outcomes) Project Promotes Cross-Sector Collaboration and Evidence-Based Trauma-Informed Care*; Pediatrics, 2021;
26. Cadiz, S.; Savage, A.; Bonavota, D.; Hollywood, J.; Butters, E.; Neary, M.; Quiros, L. The Portal Project: A Layered Approach to Integrating Trauma into Alcohol and Other Drug Treatment for Women. *Alcohol. Treat. Q.* **2005**, *22*, 121–139, doi:10.1300/J020v22n03_07.
27. Cannon, L.M.; Coolidge, E.M.; LeGierse, J.; Moskowitz, Y.; Buckley, C.; Chapin, E.; Warren, M.; Kuzma, E.K. Trauma-Informed Education: Creating and Pilot Testing a Nursing Curriculum on Trauma-Informed Care. *Nurse Educ. Today* **2020**, *85*, 104256, doi:10.1016/j.nedt.2019.104256.
28. Cascio, K.A. Providing Trauma-Informed Care to Women Exiting Prostitution: Assessing Programmatic Responses to Severe Trauma. *J. Trauma Dissociation* **2019**, *20*, 100–113, doi:10.1080/15299732.2018.1502713.
29. Chandler, G. From Traditional Inpatient to Trauma-Informed Treatment: Transferring Control From Staff to Patient. *J. Am. Psychiatr. Nurses Assoc.* **2008**, *14*, 363–371, doi:10.1177/1078390308326625.
30. Chandler, G.E. Reducing Use of Restraints and Seclusion to Create a Culture of Safety. *J. Psychosoc. Nurs. Ment. Health Serv.* **2012**, *50*, 29–36, doi:10.3928/02793695-20120906-97.
31. Choi, K.R.; Seng, J.S. Pilot for Nurse-Led, Interprofessional In-Service Training on Trauma-Informed Perinatal Care. *J. Contin. Educ. Nurs.* **2015**, *46*, 515–521, doi:10.3928/00220124-20151020-04.

32. Chokshi, B.; Walsh, K.; Dooley, D.; Falusi, O.; Deyton, L.; Beers, L. Teaching Trauma-Informed Care: A Symposium for Medical Students. *MedEdPORTAL* **2020**, 11061, doi:10.15766/mep_2374-8265.11061.
33. Chowdhury, D. Developing a Trauma-Informed Workforce for the Opioid Crisis in a Rural Community in the United States: A Case Study. *J. Ment. Health Train. Educ. Pract.* **2022**, *17*, 12–26, doi:10.1108/JMHTEP-06-2021-0070.
34. Christian-Brandt, A.S.; Santacrose, D.E.; Barnett, M.L. In the Trauma-Informed Care Trenches: Teacher Compassion Satisfaction, Secondary Traumatic Stress, Burnout, and Intent to Leave Education within Underserved Elementary Schools. *Child Abuse Negl.* **2020**, *110*, 104437, doi:10.1016/j.chiabu.2020.104437.
35. Chung, S.; Domino, M.E.; Morrissey, J.P. Changes in Treatment Content of Services During Trauma-Informed Integrated Services for Women with Co-Occurring Disorders. *Community Ment. Health J.* **2009**, *45*, 375–384, doi:10.1007/s10597-009-9192-9.
36. Cilia Vincenti, S.; Grech, P.; Scerri, J. Psychiatric Hospital Nurses' Attitudes towards Trauma-informed Care. *J. Psychiatr. Ment. Health Nurs.* **2022**, *29*, 75–85, doi:10.1111/jpm.12747.
37. Clements, A.D.; Haas, B.; Cyphers, N.A.; Hoots, V.; Barnet, J. Creating a Communitywide System of Trauma-Informed Care. *Prog. Community Health Partnersh. Res. Educ. Action* **2020**, *14*, 499–507, doi:10.1353/cpr.2020.0055.
38. Collin-Vézina, D.; Brend, D.; Black, K.; Beeman, I.; Brown, S. Impacts of Child Welfare Worker and Clientele Characteristics on Attitudes toward Trauma Informed-Care. *Dev. Child Welf.* **2020**, *2*, 244–261, doi:10.1177/2516103220963139.
39. Connell, C.M.; Lang, J.M.; Zorba, B.; Stevens, K. Enhancing Capacity for Trauma-informed Care in Child Welfare: Impact of a Statewide Systems Change Initiative. *Am. J. Community Psychol.* **2019**, *64*, 467–480, doi:10.1002/ajcp.12375.
40. Conners-Burrow, N.A.; Kramer, T.L.; Sigel, B.A.; Helpenstill, K.; Sievers, C.; McKelvey, L. Trauma-Informed Care Training in a Child Welfare System: Moving It to the Front Line. *Child. Youth Serv. Rev.* **2013**, *35*, 1830–1835, doi:10.1016/j.childyouth.2013.08.013.
41. Conradi, L.; Agosti, J.; Tullberg, E.; Richardson, L.; Langan, H.; Ko, S.; Wilson, C. Promising Practices and Strategies for Using Trauma-Informed Child Welfare Practice to Improve Foster Care Placement Stability: A Breakthrough Series Collaborative. *Child Welfare* **2011**, *90*, 207–225.
42. Crosby, R.G.; Smith, E.I.; Gage, J.; Blanchette, L. Trauma-Informed Children's Ministry: A Qualitative Descriptive Study. *J. Child Adolesc. Trauma* **2021**, *14*, 493–505, doi:10.1007/s40653-020-00334-w.
43. Damian, A.J.; Gallo, J.; Leaf, P.; Mendelson, T. Organizational and Provider Level Factors in Implementation of Trauma-Informed Care after a City-Wide Training: An Explanatory Mixed Methods Assessment. *BMC Health Serv. Res.* **2017**, *17*, 750, doi:10.1186/s12913-017-2695-0.
44. Damian, A.J.; Mendelson, T.; Bowie, J.; Gallo, J.J. A Mixed Methods Exploratory Assessment of the Usefulness of Baltimore City Health Department's Trauma-Informed Care Training Intervention. *Am. J. Orthopsychiatry* **2019**, *89*, 228–236, doi:10.1037/ort0000357.
45. Désilets, L.; Fernet, M.; Otis, J.; Cousineau, M.-M.; Massie, L.; De Pokomandy, A.; Nengeh Mensah, M. Trauma-Informed Practices to Address Intersections Between HIV and Intimate Partner Violence Among Women: Perspective of Community Service Providers. *J. Assoc. Nurses AIDS Care* **2020**, *31*, 176–189, doi:10.1097/JNC.0000000000000163.
46. Dichter, M.E.; Teitelman, A.; Klusaritz, H.; Maurer, D.M.; Cronholm, P.F.; Doubeni, C.A. Trauma-Informed Care Training in Family Medicine Residency Programs: Results From a CERA Survey. *Fam. Med.* **2018**, *50*, 617–622, doi:10.22454/FamMed.2018.505481.
47. Diebold, J.; Sperlich, M.; Heagle, E.; Marris, W.; Green, S. *Trauma Talks* : Exploring Personal Narratives of Trauma-Informed Care through Podcasting. *J. Technol. Hum. Serv.* **2021**, *39*, 1–23, doi:10.1080/15228835.2020.1820425.
48. Diggins, J. Reductions in Behavioural and Emotional Difficulties from a Specialist, Trauma-Informed School. *Educ. Dev. Psychol.* **2021**, *38*, 194–205, doi:10.1080/20590776.2021.1923131.

49. Donisch, K.; Bray, C.; Gewirtz, A. Child Welfare, Juvenile Justice, Mental Health, and Education Providers' Conceptualizations of Trauma-Informed Practice. *Child Maltreat.* **2016**, *21*, 125–134, doi:10.1177/1077559516633304.
50. Doughty, K. Increasing Trauma-Informed Awareness and Practice in Higher Education. *J. Contin. Educ. Health Prof.* **2020**, *40*, 66–68, doi:10.1097/CEH.0000000000000279.
51. Douglass, A.; Chickerella, R.; Maroney, M. Becoming Trauma-Informed: A Case Study of Early Educator Professional Development and Organizational Change. *J. Early Child. Teach. Educ.* **2021**, *42*, 182–202, doi:10.1080/10901027.2021.1918296.
52. Drabble, L.A.; Jones, S.; Brown, V. Advancing Trauma-Informed Systems Change in a Family Drug Treatment Court Context. *J. Soc. Work Pract. Addict.* **2013**, *13*, 91–113, doi:10.1080/1533256X.2012.756341.
53. Dueweke, A.R.; Hanson, R.F.; Wallis, E.; Fanguy, E.; Newman, C. Training Pediatric Primary Care Residents in Trauma-Informed Care: A Feasibility Trial. *Clin. Pediatr. (Phila.)* **2019**, *58*, 1239–1249, doi:10.1177/0009922819859868.
54. Edwards, K.M.; Mullet, N.; Siller, L. Trauma Informed Practices of a Sober Living Home for Women with Addiction and Victimization Histories. *J. Soc. Work Pract. Addict.* **2023**, *23*, 102–115, doi:10.1080/1533256X.2021.2004354.
55. Elwyn, L.J.; Esaki, N.; Smith, C.A. Importance of Leadership and Employee Engagement in Trauma-Informed Organizational Change at a Girls' Juvenile Justice Facility. *Hum. Serv. Organ. Manag. Leadersh. Gov.* **2017**, *41*, 106–118, doi:10.1080/23303131.2016.1200506.
56. Espelage, D.L.; Valido, A.; El Sheikh, A.J.; Robinson, L.E.; Ingram, K.M.; Torgal, C.; Atria, C.G.; Chalfant, P.K.; Nicholson, A.M.; Salama, C.D.; et al. Pilot Evaluation of K-12 School Security Professionals Online Training: Understanding Trauma and Social–Emotional Learning. *School Ment. Health* **2021**, *13*, 41–54, doi:10.1007/s12310-020-09399-2.
57. Eyal, M.; Bauer, T.; Playfair, E.; McCarthy, C.J. Mind-Body Group for Teacher Stress: A Trauma-Informed Intervention Program. *J. Spec. Group Work* **2019**, *44*, 204–221, doi:10.1080/01933922.2019.1634779.
58. Farro, S.A.; Clark, C.; Hopkins Eyles, C. Assessing Trauma-Informed Care Readiness in Behavioral Health: An Organizational Case Study. *J. Dual Diagn.* **2011**, *7*, 228–241, doi:10.1080/15504263.2011.620429.
59. Fragkiadaki, E.; Ghafoori, B.; Triliva, S.; Sfakianaki, R. A Pilot Study of a Trauma Training for Healthcare Workers Serving Refugees in Greece: Perceptions of Feasibility of Task-Shifting Trauma Informed Care. *J. Aggress. Maltreatment Trauma* **2020**, *29*, 442–460, doi:10.1080/10926771.2019.1662866.
60. Fraser, J.G.; Griffin, J.L.; Barto, B.L.; Lo, C.; Wenz-Gross, M.; Spinazzola, J.; Bodian, R.A.; Nisenbaum, J.M.; Bartlett, J.D. Implementation of a Workforce Initiative to Build Trauma-Informed Child Welfare Practice and Services: Findings from the Massachusetts Child Trauma Project. *Child. Youth Serv. Rev.* **2014**, *44*, 233–242, doi:10.1016/j.childyouth.2014.06.016.
61. Galvin, E.; O'Donnell, R.; Mousa, A.; Halfpenny, N.; Skouteris, H. Attitudes towards Trauma-Informed Care in Residential out-of-Home Care. *Child. Youth Serv. Rev.* **2020**, *117*, 105346, doi:10.1016/j.childyouth.2020.105346.
62. Gilmer, T.P.; Center, K.; Casteel, D.; Choi, K.; Innes-Gomberg, D.; Lansing, A.E. Developing Trauma Resilient Communities through Community Capacity-Building. *BMC Public Health* **2021**, *21*, 1681, doi:10.1186/s12889-021-11723-7.
63. Goetz, S.B.; Taylor-Trujillo, A. A Change in Culture: Violence Prevention in an Acute Behavioral Health Setting. *J. Am. Psychiatr. Nurses Assoc.* **2012**, *18*, 96–103, doi:10.1177/1078390312439469.
64. Goldstein, E.; Murray-García, J.; Sciolla, A.F.; Topitzes, J. Medical Students' Perspectives on Trauma-Informed Care Training. *Perm. J.* **2018**, *22*, 17–126, doi:10.7812/TPP/17-126.

65. Greenwald, R.; Maguin, E.; Smyth, N.J.; Greenwald, H.; Johnston, K.G.; Weiss, R.L. Teaching Trauma-Related Insight Improves Attitudes and Behaviors toward Challenging Clients. *Traumatology* **2008**, *14*, 1–11, doi:10.1177/1534765608315635.
66. Guevara, A.M.M.; Johnson, S.L.; Elam, K.; Rivas, T.; Berendzen, H.; Gal-Szabo, D.E. What Does It Mean to Be Trauma-Informed? A Multi-System Perspective from Practitioners Serving the Community. *J. Child Fam. Stud.* **2021**, *30*, 2860–2876, doi:10.1007/s10826-021-02094-z.
67. Hale, R.; Wendler, M.C. Evidence-Based Practice: Implementing Trauma-Informed Care of Children and Adolescents in the Inpatient Psychiatric Setting. *J. Am. Psychiatr. Nurses Assoc.* **2023**, *29*, 161–170, doi:10.1177/1078390320980045.
68. Hall, A.; McKenna, B.; Dearie, V.; Maguire, T.; Charleston, R.; Furness, T. Educating Emergency Department Nurses about Trauma Informed Care for People Presenting with Mental Health Crisis: A Pilot Study. *BMC Nurs.* **2016**, *15*, 21, doi:10.1186/s12912-016-0141-y.
69. Hawkins, B.E.; Coupet, E.; Saint-Hilaire, S.; Dodington, J. Trauma-Informed Acute Care of Patients With Violence-Related Injury. *J. Interpers. Violence* **2022**, *37*, NP18376–NP18393, doi:10.1177/08862605211041375.
70. Hickle, K. Introducing a Trauma-informed Capability Approach in Youth Services. *Child. Soc.* **2020**, *34*, 537–551, doi:10.1111/chso.12388.
71. Hodgdon, H.B.; Kinniburgh, K.; Gabowitz, D.; Blaustein, M.E.; Spinazzola, J. Development and Implementation of Trauma-Informed Programming in Youth Residential Treatment Centers Using the ARC Framework. *J. Fam. Violence* **2013**, *28*, 679–692, doi:10.1007/s10896-013-9531-z.
72. Hunsley, J.; Razuri, E.; Ninziza Kamanzi, D.; Sullivan, H.; Call, C.; Styffe, E.; Hategekimana, C. Experiences of Lay Social Workers Trained in a Trauma-Informed Intervention in the Deinstitutionalization of Rwanda. *J. Child. Serv.* **2021**, *16*, 289–303, doi:10.1108/JCS-09-2020-0056.
73. Im, H.; Swan, L.E.T. Capacity Building for Refugee Mental Health in Resettlement: Implementation and Evaluation of Cross-Cultural Trauma-Informed Care Training. *J. Immigr. Minor. Health* **2020**, *22*, 923–934, doi:10.1007/s10903-020-00992-w.
74. Im, H.; Swan, L.E.T. Working towards Culturally Responsive Trauma-Informed Care in the Refugee Resettlement Process: Qualitative Inquiry with Refugee-Serving Professionals in the United States. *Behav. Sci.* **2021**, *11*, 155, doi:10.3390/bs11110155.
75. Isobel, S.; Edwards, C. Using Trauma Informed Care as a Nursing Model of Care in an Acute Inpatient Mental Health Unit: A Practice Development Process. *Int. J. Ment. Health Nurs.* **2017**, *26*, 88–94, doi:10.1111/inm.12236.
76. Isobel, S.; Delgado, C. Safe and Collaborative Communication Skills: A Step towards Mental Health Nurses Implementing Trauma Informed Care. *Arch. Psychiatr. Nurs.* **2018**, *32*, 291–296, doi:10.1016/j.apnu.2017.11.017.
77. Jankowski, M.K.; Schifferdecker, K.E.; Butcher, R.L.; Foster-Johnson, L.; Barnett, E.R. Effectiveness of a Trauma-Informed Care Initiative in a State Child Welfare System: A Randomized Study. *Child Maltreat.* **2019**, *24*, 86–97, doi:10.1177/1077559518796336.
78. Keesler, J.M. Trauma-informed Day Services for Individuals with Intellectual/Developmental Disabilities: Exploring Staff Understanding and Perception within an Innovative Programme. *J. Appl. Res. Intellect. Disabil.* **2016**, *29*, 481–492, doi:10.1111/jar.12197.
79. Keesler, J.M.; Isham, C. Trauma-Informed Day Services: An Initial Conceptualization and Preliminary Assessment. *J. Policy Pract. Intellect. Disabil.* **2017**, *14*, 164–175, doi:10.1111/jppi.12206.
80. Keesler, J.M. From the DSP Perspective: Exploring the Use of Practices That Align With Trauma-Informed Care in Organizations Serving People With Intellectual and Developmental Disabilities. *Intellect. Dev. Disabil.* **2020**, *58*, 208–220, doi:10.1352/1934-9556-58.3.208.
81. Keesler, J.M. Promoting Satisfaction and Reducing Fatigue: Understanding the Impact of Trauma-informed Organizational Culture on Psychological Wellness among Direct Service Providers. *J. Appl. Res. Intellect. Disabil.* **2020**, *33*, 939–949, doi:10.1111/jar.12715.
82. Kenny, M.C.; Vazquez, A.; Long, H.; Thompson, D. Implementation and Program Evaluation of Trauma-Informed Care Training across State Child Advocacy Centers: An Exploratory Study. *Child. Youth Serv. Rev.* **2017**, *73*, 15–23, doi:10.1016/j.childyouth.2016.11.030.

83. Kerns, S.E.U.; Pullmann, M.D.; Negrete, A.; Uomoto, J.A.; Berliner, L.; Shogren, D.; Silverman, E.; Putnam, B. Development and Implementation of a Child Welfare Workforce Strategy to Build a Trauma-Informed System of Support for Foster Care. *Child Maltreat.* **2016**, *21*, 135–146, doi:10.1177/1077559516633307.
84. Kim, S.; Crooks, C.V.; Bax, K.; Shokoohi, M. Impact of Trauma-Informed Training and Mindfulness-Based Social–Emotional Learning Program on Teacher Attitudes and Burnout: A Mixed-Methods Study. *School Ment. Health* **2021**, *13*, 55–68, doi:10.1007/s12310-020-09406-6.
85. Kim, J.; Aggarwal, A.; Maloney, S.; Tibbits, M. Organizational Assessment to Implement Trauma-Informed Care for First Responders, Child Welfare Providers, and Healthcare Professionals. *Prof. Psychol. Res. Pract.* **2021**, *52*, 569–578, doi:10.1037/pro0000408.
86. Knaak, S.; Sandrelli, M.; Patten, S. How a Shared Humanity Model Can Improve Provider Well-Being and Client Care: An Evaluation of Fraser Health's Trauma and Resiliency Informed Practice (TRIP) Training Program. *Healthc. Manage. Forum* **2021**, *34*, 87–92, doi:10.1177/0840470420970594.
87. Kokokyi, S.; Klest, B.; Anstey, H. A Patient-Oriented Research Approach to Assessing Patients' and Primary Care Physicians' Opinions on Trauma-Informed Care. *PLOS ONE* **2021**, *16*, e0254266, doi:10.1371/journal.pone.0254266.
88. Kramer, T.L.; Sigel, B.A.; Conners-Burrow, N.A.; Savary, P.E.; Tempel, A. A Statewide Introduction of Trauma-Informed Care in a Child Welfare System. *Child. Youth Serv. Rev.* **2013**, *35*, 19–24, doi:10.1016/j.childyouth.2012.10.014.
89. Kramer, M.G. Sanctuary in a Residential Treatment Center: Creating a Therapeutic Community of Hope Countering Violence. *Ther. Communities Int. J. Ther. Communities* **2016**, *37*, 69–83, doi:10.1108/TC-01-2015-0005.
90. Krishnamoorthy, G.; Ayre, K. Sustaining Interdisciplinary Work in Trauma-Informed Education. *Aust. Educ. Res.* **2022**, *49*, 529–546, doi:10.1007/s13384-021-00483-3.
91. Kuhn, T.M.; Cyperski, M.A.; Shaffer, A.M.; Gracey, K.A.; Adams, M.K.; Billings, G.M.; Ebert, J.S. Installing Trauma-Informed Care through the Tennessee Child Protective Services Academy. *Psychol. Serv.* **2019**, *16*, 143–152, doi:10.1037/ser0000320.
92. Kusmaul, N.; Wilson, B.; Nochajski, T. The Infusion of Trauma-Informed Care in Organizations: Experience of Agency Staff. *Hum. Serv. Organ. Manag. Leadersh. Gov.* **2015**, *39*, 25–37, doi:10.1080/23303131.2014.968749.
93. Kusmaul, N.; Wolf, M.R.; Sahoo, S.; Green, S.A.; Nochajski, T.H. Client Experiences of Trauma-Informed Care in Social Service Agencies. *J. Soc. Serv. Res.* **2019**, *45*, 589–599, doi:10.1080/01488376.2018.1481178.
94. Lang, J.M.; Campbell, K.; Shanley, P.; Crusto, C.A.; Connell, C.M. Building Capacity for Trauma-Informed Care in the Child Welfare System: Initial Results of a Statewide Implementation. *Child Maltreat.* **2016**, *21*, 113–124, doi:10.1177/1077559516635273.
95. Lauridsen, M.B.; Munkejord, M.C. Creating Conditions for Professional Development through a Trauma-Informed and Restorative Practice. *Soc. Work* **2022**, *67*, 135–144, doi:10.1093/sw/swac005.
96. Levine, S.; Varcoe, C.; Browne, A.J. "We Went as a Team Closer to the Truth": Impacts of Interprofessional Education on Trauma- and Violence-Informed Care for Staff in Primary Care Settings. *J. Interprof. Care* **2021**, *35*, 46–54, doi:10.1080/13561820.2019.1708871.
97. Liang, C.T.H.; Liu, L.; Rocchino, G.H.; Kohler, B.A.; Rosenberger, T. Trauma-Informed Care Training for Educators: Some Preliminary Evidence. *J. Prev. Health Promot.* **2020**, *1*, 240–263, doi:10.1177/2632077020972038.
98. Linn, N.; Stephens, K.; Swanson-Biearman, B.; Lewis, D.; Whiteman, K. Implementing Trauma-Informed Strategies for Mothers of Infants with Neonatal Abstinence Syndrome. *MCN Am. J. Matern. Nurs.* **2021**, *46*, 211–216, doi:10.1097/NMC.0000000000000728.
99. Loomis, B.; Epstein, K.; Dauria, E.F.; Dolce, L. Implementing a Trauma-Informed Public Health System in San Francisco, California. *Health Educ. Behav.* **2019**, *46*, 251–259, doi:10.1177/1090198118806942.

100. Loomis, A.M.; Felt, F. Knowledge, Skills, and Self-Reflection: Linking Trauma Training Content to Trauma-Informed Attitudes and Stress in Preschool Teachers and Staff. *School Ment. Health* **2021**, *13*, 101–113, doi:10.1007/s12310-020-09394-7.
101. Lotzin, A.; Buth, S.; Sehner, S.; Hiller, P.; Martens, M.-S.; Pawils, S.; Metzner, F.; Read, J.; Härter, M.; Schäfer, I.; et al. "Learning How to Ask": Effectiveness of a Training for Trauma Inquiry and Response in Substance Use Disorder Healthcare Professionals. *Psychol. Trauma Theory Res. Pract. Policy* **2018**, *10*, 229–238, doi:10.1037/tra0000269.
102. Lotzin, A.; Buth, S.; Sehner, S.; Hiller, P.; Pawils, S.; Metzner, F.; Read, J.; Härter, M.; Schäfer, I. Reducing Barriers to Trauma Inquiry in Substance Use Disorder Treatment – a Cluster-Randomized Controlled Trial. *Subst. Abuse Treat. Prev. Policy* **2019**, *14*, 23, doi:10.1186/s13011-019-0211-8.
103. Lotzin, A.; Buth, S.; Sehner, S.; Hiller, P.; Martens, M.-S.; Read, J.; Härter, M.; Cowlishaw, S.; Schäfer, I. Learning How to Ask - Does a One-Day Training Increase Trauma Inquiry in Routine Substance Use Disorder Practice? Results of a Cluster-Randomized Controlled Trial. *J. Subst. Abuse Treat.* **2019**, *107*, 8–16, doi:10.1016/j.jsat.2019.08.005.
104. Müller, R.; Kenney, M. A Science of Hope? Tracing Emergent Entanglements between the Biology of Early Life Adversity, Trauma-Informed Care, and Restorative Justice. *Sci. Technol. Hum. Values* **2021**, *46*, 1230–1260, doi:10.1177/0162243920974095.
105. MacLochlainn, J.; Kirby, K.; McFadden, P.; Mallett, J. An Evaluation of Whole-School Trauma-Informed Training Intervention Among Post-Primary School Personnel: A Mixed Methods Study. *J. Child Adolesc. Trauma* **2022**, *15*, 925–941, doi:10.1007/s40653-021-00432-3.
106. Mantler, T.; Wolfe, B. Evaluation of Trauma-Informed Integrated Health Models of Care for Women: A Qualitative Case Study Approach. *Partn. Abuse* **2018**, *9*, 118–136, doi:10.1891/1946-6560.9.2.118.
107. Marvin, A.F.; Volino Robinson, R. Implementing Trauma-Informed Care at a Non-Profit Human Service Agency in Alaska: Assessing Knowledge, Attitudes, and Readiness for Change. *J. Evid.-Inf. Soc. Work* **2018**, *15*, 550–563, doi:10.1080/23761407.2018.1489324.
108. Matte-Landry, A.; Collin-Vézina, D. Patterns of Change in Restraints, Seclusions and Time-Outs over the Implementation of Trauma-Informed Staff Training Programs in Residential Care for Children and Youth. *Resid. Treat. Child. Youth* **2022**, *39*, 154–178, doi:10.1080/0886571X.2021.1929660.
109. McEvedy, S.; Maguire, T.; Furness, T.; McKenna, B. Sensory Modulation and Trauma-Informed-Care Knowledge Transfer and Translation in Mental Health Services in Victoria: Evaluation of a Statewide Train-the-Trainer Intervention. *Nurse Educ. Pract.* **2017**, *25*, 36–42, doi:10.1016/j.nepr.2017.04.012.
110. McIntyre, E.M.; Baker, C.N.; Overstreet, S.; The New Orleans Trauma-Informed Schools Learning Collaborative Evaluating Foundational Professional Development Training for Trauma-Informed Approaches in Schools. *Psychol. Serv.* **2019**, *16*, 95–102, doi:10.1037/ser0000312.
111. McNamara, M.; Cane, R.; Hoffman, Y.; Reese, C.; Schwartz, A.; Stolbach, B. Training Hospital Personnel in Trauma-Informed Care: Assessing an Interprofessional Workshop With Patients as Teachers. *Acad. Pediatr.* **2021**, *21*, 158–164, doi:10.1016/j.acap.2020.05.019.
112. Minne, E.P.; Gorelik, G. The Protective Role of Trauma Informed Attitudes on Perceived Stress Among Teachers and School Staff. *J. Child Adolesc. Trauma* **2022**, *15*, 275–283, doi:10.1007/s40653-021-00389-3.
113. Mueller, S.; Hart, M.; Carr, C. Resilience Building Programs in U.S. Corrections Facilities: An Evaluation of Trauma-Informed Practices in Place. *J. Aggress. Maltreatment Trauma* **2023**, *32*, 242–261, doi:10.1080/10926771.2021.2008082.
114. Murphy, K.; Moore, K.A.; Redd, Z.; Malm, K. Trauma-Informed Child Welfare Systems and Children's Well-Being: A Longitudinal Evaluation of KVC's Bridging the Way Home Initiative. *Child. Youth Serv. Rev.* **2017**, *75*, 23–34, doi:10.1016/j.childyouth.2017.02.008.
115. Nation, L.; Spence, N.; Parker, S.; Wheeler, M.P.; Powe, K.; Siew, M.; Nevin, T.; McKay, M.; White, M.; Dark, F.L. Implementing Introductory Training in Trauma-Informed Care Into Mental Health Rehabilitation Services: A Mixed Methods Evaluation. *Front. Psychiatry* **2022**, *12*, 810814, doi:10.3389/fpsyt.2021.810814.

116. Niimura, J.; Nakanishi, M.; Okumura, Y.; Kawano, M.; Nishida, A. Effectiveness of 1-day Trauma-informed Care Training Programme on Attitudes in Psychiatric Hospitals: A Pre–Post Study. *Int. J. Ment. Health Nurs.* **2019**, *28*, 980–988, doi:10.1111/inm.12603.
117. Orapallo, A.; Grant, B.-J.; Baker, C.N. Examining the Effectiveness of Trauma Smart® Training: Staff Satisfaction, Knowledge, and Attitudes. *Psychol. Trauma Theory Res. Pract. Policy* **2021**, *13*, 891–898, doi:10.1037/tra0001075.
118. Palfrey, N.; Reay, R.E.; Aplin, V.; Cubis, J.C.; McAndrew, V.; Riordan, D.M.; Raphael, B. Achieving Service Change Through the Implementation of a Trauma-Informed Care Training Program Within a Mental Health Service. *Community Ment. Health J.* **2019**, *55*, 467–475, doi:10.1007/s10597-018-0272-6.
119. Parry, S.L.; Williams, T.; Burbidge, C. Restorative Parenting: Delivering Trauma-Informed Residential Care for Children in Care. *Child Youth Care Forum* **2021**, *50*, 991–1012, doi:10.1007/s10566-021-09610-8.
120. Piper, K.N.; Brown, L.L.; Tamler, I.; Kalokhe, A.S.; Sales, J.M. Application of the Consolidated Framework for Implementation Research to Facilitate Delivery of Trauma-Informed HIV Care. *Ethn. Dis.* **2021**, *31*, 109–118, doi:10.18865/ed.31.1.109.
121. Poldon, S.; Duhn, L.; Camargo Plazas, P.; Purkey, E.; Tranmer, J. Exploring How Sexual Assault Nurse Examiners Practise Trauma-Informed Care. *J. Forensic. Nurs.* **2021**, *17*, 235–243, doi:10.1097/JFN.0000000000000338.
122. Putkonen, A.; Kuivalainen, S.; Louheranta, O.; Repo-Tiihonen, E.; Ryynänen, O.-P.; Kautiainen, H.; Tiihonen, J. Cluster-Randomized Controlled Trial of Reducing Seclusion and Restraint in Secured Care of Men With Schizophrenia. *Psychiatr. Serv.* **2013**, *64*, 850–855, doi:10.1176/appi.ps.201200393.
123. Ramadurai, D.; Knoeckel, J.; Stace, R.J.; Stella, S. Feasibility and Impact of Trauma-Informed Care Training in Internal Medicine Residency: A Pilot Study. *Cureus* **2022**, doi:10.7759/cureus.22368.
124. Reece, J. Planning for Youth Emotional Health in Unruly Environments: Bringing a Trauma Informed Community Building Lens to Therapeutic Planning. *Projections* **2020**, doi:10.1162/00c13b77.29ae0baa.
125. Reid, N.; Kron, A.; Rajakulendran, T.; Kahan, D.; Noble, A.; Stergiopoulos, V. Promoting Wellness and Recovery of Young Women Experiencing Gender-Based Violence and Homelessness: The Role of Trauma-Informed Health Promotion Interventions. *Violence Women* **2021**, *27*, 1297–1316, doi:10.1177/1077801220923748.
126. Riahi, S.; Dawe, I.C.; Stuckey, M.I.; Klassen, P.E. Implementation of the Six Core Strategies for Restraint Minimization in a Specialized Mental Health Organization. *J. Psychosoc. Nurs. Ment. Health Serv.* **2016**, *54*, 32–39, doi:10.3928/02793695-20160920-06.
127. Richmond, E.; McColm, R.; McCaig, M.; Binnie, V. Improving Outcomes for Trauma-Experienced Individuals through the Delivery of Trauma Awareness Training for Multi-Organisational Public Sector Workers. *J. Ment. Health Train. Educ. Pract.* **2022**, *17*, 27–35, doi:10.1108/JMHTEP-04-2021-0038.
128. Robey, N.; Margolies, S.; Sutherland, L.; Rupp, C.; Black, C.; Hill, T.; Baker, C.N. Understanding Staff- and System-Level Contextual Factors Relevant to Trauma-Informed Care Implementation. *Psychol. Trauma Theory Res. Pract. Policy* **2021**, *13*, 249–257, doi:10.1037/tra0000948.
129. Rodger, S.; Bird, R.; Hibbert, K.; Johnson, A.M.; Specht, J.; Wathen, C.N. Initial Teacher Education and Trauma and Violence Informed Care in the Classroom: Preliminary Results from an Online Teacher Education Course. *Psychol. Sch.* **2020**, *57*, 1798–1814, doi:10.1002/pits.22373.
130. Salloum, A.; Choi, M.J.; Stover, C.S. Exploratory Study on the Role of Trauma-Informed Self-Care on Child Welfare Workers' Mental Health. *Child. Youth Serv. Rev.* **2019**, *101*, 299–306, doi:10.1016/j.childyouth.2019.04.013.
131. Scheer, J.R.; Poteat, V.P. Trauma-Informed Care and Health Among LGBTQ Intimate Partner Violence Survivors. *J. Interpers. Violence* **2021**, *36*, 6670–6692, doi:10.1177/0886260518820688.

132. Schmid, M.; Lüdtke, J.; Dolitzsch, C.; Fischer, S.; Eckert, A.; Fegert, J.M. Effect of Trauma-Informed Care on Hair Cortisol Concentration in Youth Welfare Staff and Client Physical Aggression towards Staff: Results of a Longitudinal Study. *BMC Public Health* **2020**, *20*, 21, doi:10.1186/s12889-019-8077-2.
133. Serrata, J.V.; Rodriguez, R.; Castro, J.E.; Hernandez-Martinez, M. Well-Being of Latina Survivors of Intimate Partner Violence and Sexual Assault Receiving Trauma-Informed and Culturally-Specific Services. *J. Fam. Violence* **2020**, *35*, 169–180, doi:10.1007/s10896-019-00049-z.
134. Shamaskin-Garroway, A.M.; McLaughlin, E.A.; Quinn, N.; Buono, F.D. Trauma-informed Primary Care for Medical Residents. *Clin. Teach.* **2020**, *17*, 200–204, doi:10.1111/tct.13082.
135. Shier, M.L.; Turpin, A. A Multi-Dimensional Conceptual Framework for Trauma-Informed Practice in Addictions Programming. *J. Soc. Serv. Res.* **2017**, *43*, 609–623, doi:10.1080/01488376.2017.1364318.
136. Shier, M.L.; Turpin, A. Trauma-Informed Organizational Dynamics and Client Outcomes in Concurrent Disorder Treatment. *Res. Soc. Work Pract.* **2022**, *32*, 92–105, doi:10.1177/10497315211013908.
137. Simons, M.; Kimble, R.; Tyack, Z. Understanding the Meaning of Trauma-Informed Care for Burns Health Care Professionals in a Pediatric Hospital: A Qualitative Study Using Interpretive Phenomenological Analysis. *Burns* **2022**, *48*, 1462–1471, doi:10.1016/j.burns.2021.10.015.
138. Steinkopf, H.; Nordanger, D.; Stige, B.; Milde, A.M. How Do Staff in Residential Care Transform Trauma-Informed Principles into Practice? A Qualitative Study from a Norwegian Child Welfare Context. *Nord. Soc. Work Res.* **2022**, *12*, 625–639, doi:10.1080/2156857X.2020.1857821.
139. Stevens, N.R.; Ziadni, M.S.; Lillis, T.A.; Gerhart, J.; Baker, C.; Hobfoll, S.E. Perceived Lack of Training Moderates Relationship between Healthcare Providers' Personality and Sense of Efficacy in Trauma-Informed Care. *Anxiety Stress Coping* **2019**, *32*, 679–693, doi:10.1080/10615806.2019.1645835.
140. Stipp, B.; Kilpatrick, L. Trust-Based Relational Intervention as a Trauma-Informed Teaching Approach. **2021**.
141. Stokes, Y.; Jacob, J.-D.; Squires, J.; Vandyk, A. Using the ARTIC-35 to Measure Nurses' Attitudes Related to Trauma-Informed Care. *J. Nurs. Meas.* **2020**, *28*, 185–199, doi:10.1891/JNM-D-18-00073.
142. Strait, J.; Bolman, T. Consideration of Personal Adverse Childhood Experiences during Implementation of Trauma-Informed Care Curriculum in Graduate Health Programs. *Perm. J.* **2017**, *21*, 16–061, doi:10.7812/TPP/16-061.
143. Sullivan, C.M.; Goodman, L.A.; Virden, T.; Strom, J.; Ramirez, R. Evaluation of the Effects of Receiving Trauma-Informed Practices on Domestic Violence Shelter Residents. *Am. J. Orthopsychiatry* **2018**, *88*, 563–570, doi:10.1037/ort0000286.
144. Sundborg, S.A. Knowledge, Principal Support, Self-Efficacy, and Beliefs Predict Commitment to Trauma-Informed Care. *Psychol. Trauma Theory Res. Pract. Policy* **2019**, *11*, 224–231, doi:10.1037/tra0000411.
145. Tompkins, C.N.E.; Neale, J. Delivering Trauma-Informed Treatment in a Women-Only Residential Rehabilitation Service: Qualitative Study. *Drugs Educ. Prev. Policy* **2018**, *25*, 47–55, doi:10.1080/09687637.2016.1235135.
146. Topitzes, J.; Grove, T.; Meyer, E.E.; Pangratz, S.M.; Sprague, C.M. Trauma-Responsive Child Welfare Services: A Mixed Methods Study Assessing Safety, Stability, and Permanency. *J. Child Custody* **2019**, *16*, 291–312, doi:10.1080/15379418.2019.1607796.
147. Truesdale, M.; Brown, M.; Taggart, L.; Bradley, A.; Paterson, D.; Sirisena, C.; Walley, R.; Karatzias, T. Trauma-informed Care: A Qualitative Study Exploring the Views and Experiences of Professionals in Specialist Health Services for Adults with Intellectual Disabilities. *J. Appl. Res. Intellect. Disabil.* **2019**, *32*, 1437–1445, doi:10.1111/jar.12634.
148. Unick, G.J.; Bassuk, E.L.; Richard, M.K.; Paquette, K. Organizational Trauma-Informed Care: Associations with Individual and Agency Factors. *Psychol. Serv.* **2019**, *16*, 134–142, doi:10.1037/ser0000299.

149. Wagner, A.C.; Bartsch, A.A.; Manganaro, M.; Monson, C.M.; Baker, C.N.; Brown, S.M. Trauma-Informed Care Training with HIV and Related Community Service Workers: Short and Long Term Effects on Attitudes. *Psychol. Serv.* **2021**, *18*, 695–702, doi:10.1037/ser0000465.
150. Walsh, K.; Benjamin, R. Using Participatory Methods to Engage Multidisciplinary Clinical Staff in the Embedding of Trauma-Informed Care and Practice Principles in a Sub-Acute Mental Health Inpatient Unit. *J. Multidiscip. Healthc.* **2020**, *Volume 13*, 485–494, doi:10.2147/JMDH.S240240.
151. Weiss, D.; Kassam-Adams, N.; Murray, C.; Kohser, K.L.; Fein, J.A.; Winston, F.K.; Marsac, M.L. Application of a Framework to Implement Trauma-Informed Care Throughout a Pediatric Health Care Network. *J. Contin. Educ. Health Prof.* **2017**, *37*, 55–60, doi:10.1097/CEH.0000000000000140.
152. Wheeler, K.; Phillips, K.E. The Development of Trauma and Resilience Competencies for Nursing Education. *J. Am. Psychiatr. Nurses Assoc.* **2021**, *27*, 322–333.
153. Williams, T.M.; Smith, G.P. Does Training Change Practice? A Survey of Clinicians and Managers One Year after Training in Trauma-Informed Care. *J. Ment. Health Train. Educ. Pract.* **2017**, *12*, 188–198, doi:10.1108/JMHTEP-02-2016-0016.
154. Wilson, B.; Nochajski, T.H. Evaluating the Impact of Trauma-Informed Care (TIC) Perspective in Social Work Curriculum. *Soc. Work Educ.* **2016**, *35*, 589–602, doi:10.1080/02615479.2016.1164840.
155. Wisdom, J.P.; Wenger, D.; Robertson, D.; Van Bramer, J.; Sederer, L.I. The New York State Office of Mental Health Positive Alternatives to Restraint and Seclusion (PARS) Project. *Psychiatr. Serv.* **2015**, *66*, 851–856, doi:10.1176/appi.ps.201400279.
156. Wojciak, A.S.; Powers, J.J.; Goslin, A.; Sandoval, J.A.; Hooper, L.M. Perceptions of We Can! Building Relationships and Resilience: A School Wide Trauma-Informed Training, from Trainers and Recipients. *Advers. Resil. Sci.* **2022**, *3*, 81–97, doi:10.1007/s42844-022-00055-z.
157. Woodside-Jiron, H.; Jorgenson, S.; Strolin-Goltzman, J.; Jorgenson, J. *“The Glue That Makes the Glitter Stick”* : Preliminary Outcomes Associated with a Trauma-Informed, Resiliency-Based, Interprofessional Graduate Course for Child Welfare, Mental Health, and Education. *J. Public Child Welf.* **2019**, *13*, 307–324, doi:10.1080/15548732.2019.1600630.
